# Development of Colloidal Gold-Based Immunochromatographic Strips for Rapid Detection and Surveillance of Japanese Encephalitis Virus in Dogs across Shanghai, China

**DOI:** 10.3390/v16020258

**Published:** 2024-02-06

**Authors:** Dengke Zhong, Abdul Wahaab, Jiayang Zheng, Junjie Zhang, Zhiyong Ma, Jianchao Wei

**Affiliations:** 1 Shanghai Vocational College of Agriculture and Forestry, Shanghai 201600, China; 211506@shafc.edu.cn; 2 Shanghai Veterinary Research Institute, Chinese Academy of Agricultural Sciences, Shanghai 200241, China; wahaaabwahaaab@gmail.com (A.W.); asd126163@163.com (J.Z.); m17317271403@163.com (J.Z.); zhiyongma@shvri.ac.cn (Z.M.); 3 Department of Entomology, Center for Infectious Disease Dynamics and The Huck Institutes of the Life Sciences, The Pennsylvania State University, University Park, PA 16801, USA

**Keywords:** Japanese encephalitis virus, EDIII, dogs, seroprevalence, immunochromatographic strip, Shanghai

## Abstract

Japanese encephalitis virus (JEV) causes acute encephalitis in humans and is of major public health concern in most Asian regions. Dogs are suitable sentinels for assessing the risk of JEV infection in humans. A neutralization test (NT) or an enzyme-linked immunosorbent assay (ELISA) is used for the serological detection of JEV in dogs; however, these tests have several limitations, and, thus, a more convenient and reliable alternative test is needed. In this study, a colloidal gold immunochromatographic strip (ICS), using a purified recombinant EDIII protein, was established for the serological survey of JEV infection in dogs. The results show that the ICSs could specifically detect JEV antibodies within 10 min without cross-reactions with antibodies against other canine viruses. The test strips could detect anti-JEV in serum with dilution up to 640 times, showing high sensitivity. The coincidence rate with the NT test was higher than 96.6%. Among 586 serum samples from dogs in Shanghai examined using the ICS test, 179 (29.98%) were found to be positive for JEV antibodies, and the high seropositivity of JEV in dogs in China was significantly correlated with the season and living environment. In summary, we developed an accurate and economical ICS for the rapid detection of anti-JEV in dog serum samples with great potential for the surveillance of JEV in dogs.

## 1. Introduction

JEV infection leads to neurological disease, and it is one of the leading viral encephalitides in the world [1]. According to World Health Organization (WHO) reports, about 24 countries in Asia and Western Pacific regions have been exposed to JEV, where it accounts for ~35,000 to 50,000 cases and 10,000 to 15,000 deaths each year [1]. However, the exact number of JEV cases probably remains under-reported [2]. The first JEV epidemics were reported in Japan in the nineteenth century [3]. JEV infections occur across a large range of Asian countries, with outbreaks occurring in Japan, China, Taiwan, Korea, the Philippines, and India [4].

JEV harbors a positive-sense RNA genome belonging to the family *Flaviviridae*. The JEV genome is approximately 11 kb in length and is proteolytically processed into three structural (Cap, prM, and E) and seven non-structural (NS1, NS2A, NS2B, NS3, NS4A, NS4B, and NS5) proteins by a complex combination of host and viral proteases [5,6,7,8]. Phylogenetically, JEV is classified into a single serotype with five genetically different genotypes (GI, GII, GIII, GIV, and GV). JEV GIII had been the most dominant strain, with several outbreaks in past. However, recent data show the emergence of the GI strain as the most common JEV genotype [9]. The JEV zoonosis life cycle contains both invertebrates (mosquitoes) and vertebrates (wild birds and pigs) [10,11]. In addition to mosquitoes as a vector, pigs and ardeid birds play the role of amplifying/reservoir hosts [12]. Recent theoretical models of vector-borne pathogen transmission show that the pathogen transmission rate mainly depends on the proportion of vector blood meals taken from competent hosts versus dead-end hosts [13]. The E protein (53–55 kDa) is a typical membrane glycoprotein, and it is responsible for a number of important processes, such as viral attachment, fusion, and virulence [13]. The ectodomain of the E protein can be separated into three structural domains: E domains I (EDI) to III (EDIII). EDIII is also involved in the binding to host receptors and contains specific epitopes that elicit neutralizing antibodies [13]. Thus, the EDIII protein could be employed as a candidate antigen for a diagnostic or subunit vaccine of JEV.

Previously, several serological surveys have been conducted on pig farms and wild boars, which tend to show high seropositivity in different regions of the world, including China [14,15,16,17,18]. As these animals live apart from human populations, serosurveys of pigs and wild animals may not indicate the prevalence of JEV in urban/residential areas. However, additional monitoring of the risk of JEV infection in humans in JEV-endemic areas can be carried out by examining seroprevalence in companion animals. Previous studies’ experimental data demonstrate that, after JEV challenge, dogs do not develop any clinical signs, or viremia, but JEV seroprevalence in dog populations, as sentinels, may be valuable in evaluating the JEV risk to humans in urban/residential areas [19,20]. All over China, people keep dogs as companion pets and to guard their property. Dogs live closest to human dwellings, and they could be exposed to arboviruses to the same extent as their owners.

Serological tests, such as the virus neutralization test (VNT), hemagglutination inhibition (HI) test, and enzyme-linked immunosorbent assay (ELISA), have been performed to detect JEV-specific antibodies in serum [21,22,23,24]. The HI test requires a large volume of serum and fresh erythrocytes, and VNT requires a special facility (e.g., biosafety level 2 or 3) and a high level of technical skill. However, specific ELISA tests have been developed and evaluated for serological surveys among humans, pigs, bats, and dogs [25,26,27,28,29]. Immunofluorescent assays (IFAs) have been developed for the detection of antibodies against JEV, and they have been effective for the diagnosis of different flaviviruses, such as Yellow fever virus and West Nile virus [30,31,32]. However, these tests have lengthy procedures, with the requirements of expensive reagents and skilled persons.

The ICS was developed for the diagnosis of contagious human diseases and has been used for the last three decades, and it has recently been introduced to veterinary fields because it is easy to use, it has a short running time (within 15–20 min), and the results can be seen with the naked eye. For example, the technique is now used to detect antigens or antibodies of animal viruses, such as avian influenza virus [33], porcine reproductive and respiratory syndrome virus [34], porcine circovirus-2 [35], and JEV [36].

To improve the JEV serosurveillance in dogs that share a living space with humans, we developed immunochromatographic strips (ICSs) based on domain III (EDIII) of the JEV envelope protein, and we successfully applied them for the surveillance of JEV antibodies in dogs in China to assess the risk of human infection with JEV. This may provide technical support for controlling the spread and prevalence of JEV.

## 2. Materials and Methods

### 2.1. Virus and Serum Samples

The JEV SA14-14-2 strain (GenBank accession no. AF315119) was propagated on BHK-21 cells, and a 50% tissue culture infective dose (TCID50) was determined for VN [37]. The JEV SA14-14-2 strain was also used as a template for the cloning and expression of the JEV recombinant EDⅢ protein in competent Escherichia coli. A total of 586 serum samples were collected from numerous pet immunization centers, hospitals, farms, and abandoned dog shelters across various districts in Shanghai in 2019–2020 for the detection of JEV antibodies.

The following were verified using VN and provided by the China Animal Health and Epidemiology Center (Shanghai Branch): serum samples positive for Japanese encephalitis virus, canine adenovirus (CAdV), canine coronavirus (CCV), canine distemper virus (CDV), canine Leptospira virus (CLV), canine parainfluenza virus (CPIV), canine parvovirus (CPV), and canine rabies virus (CRV) from experimentally infected dogs; anti-E monoclonal antibodies; and negative serum samples.

### 2.2. Cloning, Expression, and Purification of Recombinant JEV EDIII Protein

The nucleotide sequence of the EDⅢ (UniProtKB:P27395, D586-T696, a partial sequence of the JEV polyprotein) of the JEV SA14-14-2 strain was amplified via a polymerase chain reaction (PCR) with specially designed oligo primers (forward: 5′-CTAGGATCCGACAAACTGGCTCTGAA-3′; reverse: 5′-TCTCTCGAGTTACGTGCTTCCAGCTTTG-3′). The desired gene fragment was digested with the restriction endonucleases BamH I and Hind III (Takara, Dalian, China), and it was ligated into the pET-28a vector (Takara, Dalian, China). Subsequently, the recombinant pET28a-EDⅢ plasmid was transformed into *Escherichia coli* BL21-competent cells (DE3), and the resultant transformants were selected on Luria–Bertani (LB) agar plates supplemented with 50 μg/mL of kanamycin [36]. Every single colony was picked from an LB agar plate and further amplified in 5 mL of LB broth. Finally, positive clones harboring the correct insert were confirmed via PCR and sequencing. The confirmed positive clones were allowed to grow further in a 250 mL LB medium containing kanamycin (50 μg/mL) [36]. Protein expression was induced using isopropyl β-D-1-thiogalactopyranoside at a final concentration of 1.5 mM. The expressed protein was purified on a Ni column using a His-Bind purification Kit (BioRad, Hercules, CA, USA), according to the manufacturer’s instructions. The protein expression was confirmed with SDS-PAGE and Western blot, as previously described, using anti-E monoclonal antibodies [38,39].

### 2.3. Preparation of the Colloidal Gold-Labeled Suspensions

Suitable antibody concentrations for the test lines, control lines, and conjugation with the colloidal gold reagent were determined as reported in previous studies [36,40,41]. Colloidal gold particles were prepared with an improved reduction of chloroauric acid by sodium citrate, as described by Wang et al. [42]. The colloidal gold solution (pH 8.6) was mixed with purified recombinant EDⅢ proteins under electromagnetic stirring and stirred rapidly for 30 min. Bovine serum albumin (BSA) was added at a concentration of 1% to inhibit the excess reactivity of the gold colloid. The blend was centrifuged at 15,000 rpm for 1 h at 4 °C. After discarding the supernatant, the obtained conjugate pellet was resuspended in 0.2 M TBST (pH 8.6) and stored at 4 °C.

### 2.4. Preparation of the Immunochromatographic Strip

**Figure 1:**
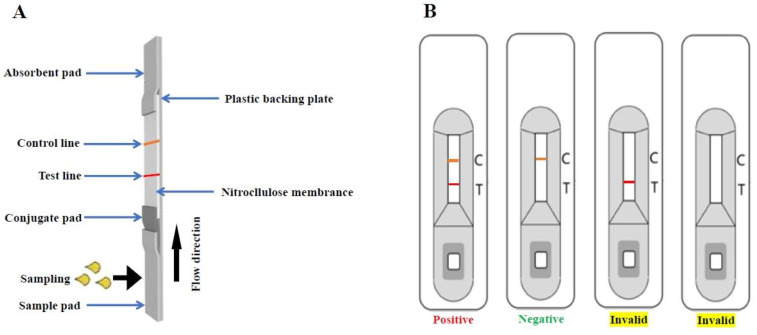
Schematic diagram of ICS. (**A**) Illustration of strip components. (**B**) Interpretation of the results using ICSs. Positive samples produce two red bands on the membrane strips; a negative sample shows only one band on the control line. If there is no colored band at all or there is one colored band only on the test line, the test is invalid. C, control line; T, test line.

The composition of the immunochromatographic strip (ICS) is shown in Figure 1, and it was prepared as follows: The ICS was divided into four compartments, i.e., an absorbent pad, a nitrocellulose membrane, a conjugate pad, and a sample pad. Staphylococcal protein A (SPA) (1.0 mg/mL Sigma, Louis, MO, USA) and anti-E monoclonal antibodies (0.1 mg/mL) were blotted on the nitrocellulose membrane and incubated for the development of a test line and a control line, respectively, using an XYZ3050 dispense workstation, and the NC membrane was then dried for 1 h at 37 °C before being stored at 4 °C. The capture test and control band were situated 0.5 cm apart in the center of the membrane. The conjugate pad, composed of a glass fiber membrane, was treated with a recombinant EDⅢ protein–colloidal gold conjugate solution and then dried under a vacuum. All components of this ICS kit were adhered to a backing plate (300 mm × 25 mm, SM31-25, Shanghai Kinbio Biotechnology Co., LTD, Shanghai, China) in proper order, as illustrated in Figure 1A. The plate was then sliced into 4 mm wide strips using an automatic cutter. Each strip was assembled on a plastic cassette (A-1, Shanghai joey Biotechnology Co., LTD, Shanghai, China) and stored at a broad temperature range (4–30 °C) before use.

### 2.5. Working Principle of Immunochromatographic Strip (ICS)

In this ICS kit, dog serum samples are diluted 100-fold with a normal saline solution and added to the sample pad. A test line will only appear if the serum sample contains JEV antibodies. When the serum samples reach the conjugate pad, the dog JEV antibodies interact with the colloidal gold JEV recombinant EDⅢ protein to form a dog JEV antibody EDⅢ–colloidal gold complex. The complex travels through the NC membrane via capillary action. When it passes through the test line, the complex reacts with SPA, resulting in a dark red band, and the excess of the antigen–antibody complex travels to the control line, where anti-dog JEV antibodies interact with the recombinant EDIII protein complex and form another red band; in this case, the results are judged as positive (Figure 1B). In contrast to this, in samples lacking JEV antibodies, the free EDⅢ–colloidal gold conjugate that cannot bind to the samples will travel to the control line. At the control line, dog anti-JEV IgG will react with the SPA, and a dark band will appear. When there is only one red band on the control line (position C), the results are considered negative; the absence of two bands (at positions C and T) suggests an invalid result. Therefore, after the addition of the serum sample, two bands will appear for positive samples within 10 min (one on the test line (position T) and one on the control line (position C)), whereas only one band will appear on the control line (position C) for negative samples (Figure 1B).

### 2.6. Specificity, Sensitivity, and Stability of the ICS

The specificity of the developed ICS was evaluated with serum samples positive for Japanese encephalitis virus, canine adenovirus, canine coronavirus, canine distemper virus, canine Leptospira virus, canine parainfluenza virus, canine parvovirus, and canine rabies virus. Anti-JEV-positive serum was used as a positive control, and negative serum was applied as a negative control.

To evaluate the sensitivity of the ICS kit, we serially diluted positive anti-JEV serum in PBS, and 50 µL of each dilution was used for the ICS test. The sensitivity of our ICS kit was determined by finding the minimum dilution concentration that produced a positive result.

The test strips were stored at room temperature (18–25 °C) and at 4 °C to determine their stability at 0, 1, 2, 3, 4, 5, 6, 7, and 8 months.

### 2.7. Seroprevalence of JEV among Dogs in Shanghai, China

A total of 586 serum samples were examined for antibodies against JEV using the developed ICSs and NT, as described previously [20,24,28,43,44]. The coincidence rate of the ICS test was compared with that of the NT [20,24,28,43,44].

### 2.8. Statistical Analysis

All data were analyzed using the Prism 5 software (GraphPad Software, La Jolla, CA, USA). All data were analyzed using a two-tailed Student’s *t*-test. *p* < 0.05 was considered statistically significant.

## 3. Results

### 3.1. Expression and Purification of the Recombinant ED3 Protein

**Figure 2:**
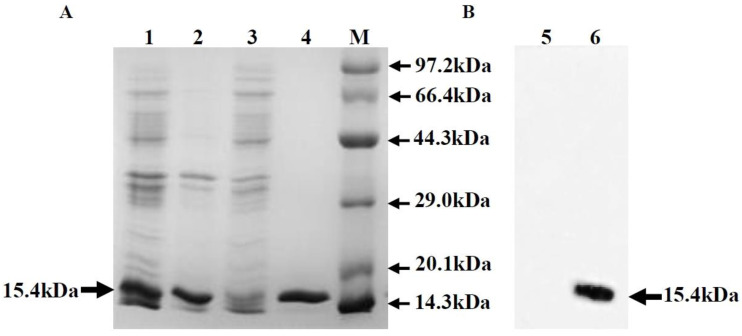
Expression and purification of the recombinant EDIII protein. (**A**) SDS-PAGE analysis of the EDIII protein expressed in *Escherichia coli*. The EDIII protein was 15.4 kDa in SDS-PAGE. The expression of pET-28a–EDIII in *E. coli* was induced with isopropyl β-d-1-thiogalactopyranoside (IPTG). Inclusion bodies were collected 16 h after induction and subjected to supersonic schizolysis. The recombinant EDIII protein was purified via affinity chromatography on a Ni^+^ spin column. Lane 1: *E. coli* containing pET-28a–EDIII induced with IPTG; Lane 2: inclusion bodies of IPTG-induced *E. coli* containing pET-28a–EDIII; Lane 3: supernatant from IPTG-induced *E. coli* containing pET-28a–EDIII; Lane 4: purified *E. coli* containing pET-28a–EDIII; M, protein marker. (**B**) Western blot analysis of the EDIII protein using anti-E monoclonal antibodies. Lane 5: uninduced *E. coli* containing pET-28a–EDIII; Lane 6: *E. coli* containing pET-28a–EDIII induced with IPTG. These protein samples were resolved electrophoretically on 12% polyacrylamide gel and transferred to a 0.2 μm polyvinylidene difluoride membrane. Membranes were treated with an anti-E monoclonal antibody followed by an HRP-conjugated goat anti-mouse IgG antibody. The reaction was visualized with a Western blot kit.

The domain III peptides from various flaviviruses, including JEV, are useful antigens for serological diagnoses [45]. The EDⅢ protein sequences of JEV were successfully cloned into the pET-28a vector, and, after IPTG induction, the JEV EDⅢ protein was expressed in competent E. coli BL21 (DE3) cells. The expressed protein was purified using Ni columns. The JEV-EDⅢ fusion protein was found mainly as inclusion bodies. The SDS-PAGE (Figure 2A: Lanes 1, 2, 3, and 4) and Western blot analyses (Figure 2B: Lines 5 and 6) demonstrated that the expressed JEV EDⅢ protein was highly purified under native conditions, with a molecular weight of 14.5 kDa, and it reacted strongly with anti-E monoclonal antibodies (Figure 2, Lane 6).

### 3.2. Specificity Evaluation of ICS

**Figure 3:**
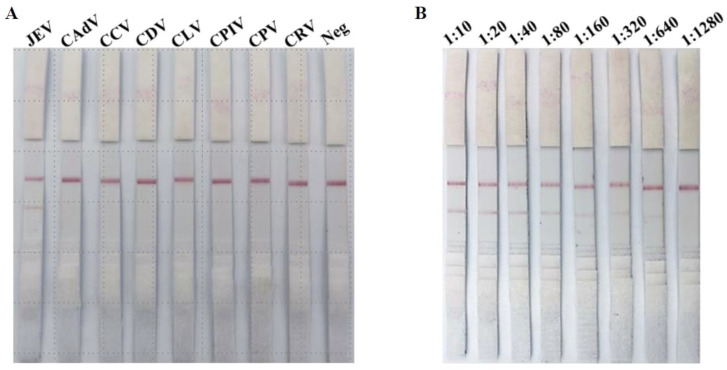
Specificity and sensitivity testing of the ICS: (**A**) Specificity of the ICS. Sera positive for different canine viruses were used to evaluate the specificity of the ICS. (**B**) Sensitivity of the ICS. JEV-positive serum was diluted from 1:10 to 1:1280 to determine the sensitivity of the ICS.

All serum samples positive for different canine viruses were used to evaluate the specificity of the ICS. Positive results were seen for dog sera containing JEV-positive antibodies, while the antisera of all other tested viruses produced negative results, as shown in Figure 3A. These data show that the ICSs are highly specific for JEV antisera and do not cross-react with other pathogenic canine viruses.

### 3.3. Sensitivity Evaluation of ICS

To determine the sensitivity of our developed ICS test, we prepared two-fold serial dilutions of the JEV-antibody-positive dog serum, i.e., 1:10, 1:20, 1:40, 1:80, 1:160, 1:320, 1:640, and 1:1280, in PBS (Figure 3B). The negative dog serum was diluted in a similar fashion for use as a negative control. No red line was observed (at position T) for the negative dog serum samples. A clear solid red line was observed for the positive serum samples (at position T) on the strips until the 1:640 dilution, indicating that the minimum detection limit is 1:640 (Figure 3B).

### 3.4. Stability Evaluation of ICS

The ICSs were evaluated for their stability to identify JEV antibodies after six months of storage at room temperature and in a refrigerator (4 °C). The strips still had the same sensitivity and specificity as the freshly produced strips after six months of storage, indicating that the ICSs have good stability.

### 3.5. Surveillance of JEV Antibodies in Dogs in Shanghai

A total of 586 dog serum samples were collected from numerous pet immunization centers, hospitals, farms, and abandoned dog shelters across various districts of Shanghai. We tested all of these serum samples by using our developed ICSs and NT. Out of the 586 samples tested, 179 (29.98%) were found to be positive for JEV antibodies. The coincidence rate of detection with these two methods was 96.6% (Table 1). This indicates that about 30% of dogs were seroconverted to JEV during the study period, which might be a public health concern.

### 3.6. Relationship between Dog JEV-Antibody-Positive Rate and Season in Shanghai

We further examined the seroprevalence of JEV during different months and seasons of the year in 2019–2020. From June to September, the environmental conditions are conducive for mosquitos’ growth in Shanghai, and it is also the epidemic period of JE [46,47,48]. We observed that the average JEV-antibody-positive ratio was 16.4% during spring (March to May), which was substantially below the average positive rate of JEV antibodies in the epidemic late autumn (46.3%) and winter seasons (35.7%) (*p* < 0.05) (Table 2). These data show that the rate of JEV antibody positivity in dogs in China has a certain seasonality. Therefore, JEV infections in dogs have some seasonal tendencies in Shanghai.

### 3.7. Relationship between Dog JEV-Antibody-Positive Rate and Living Environment in Shanghai

We further categorized the seroprevalence of JEV antibodies according to living environment. To this end, dogs were divided into three categories, i.e., domestic dogs (pet clinics and immunization centers), breeding dogs (dog farms), and stray dogs (shelters, etc.). The highest prevalence of JEV antibodies was found in stray dogs (49.5%), followed by breeding dogs (43.3%) and domestic dogs (20.6%) (*p* > 0.05). Domestic dogs had the lowest JEV antibody prevalence, possibly due to their indoor feeding behaviors and fewer outdoor activities resulting in less exposure to bites from JEV-carrying mosquitos.

## 4. Discussion

Japanese encephalitis virus (JEV) causes encephalitis and reproductive disorder in humans and pigs, respectively, having a serious impact on public health and the pig industry [1,49]. Previously, JE was considered to be mainly limited to rural areas because of the presence of rice fields, a suitable habitat for Culex mosquitoes, which play the main role as a vector [50,51], and because of the presence of the pig/bird population as a reservoir/amplification host for JEV [12,52].

However, recent data on JEV show that JEV has been isolated from local mosquitoes collected from urban areas, as well as in urban vertebrate hosts, including humans, as a result of seroconversion [53,54,55]. Past data on JEV seroconversion show that dogs can be infected with JEV and might play a role in JEV transmission [20,56]. Furthermore, dogs live in close proximity to humans, are not vaccinated against JEV, do not show symptoms when infected, and maintain virus neutralization titers for long periods after JEV infection. All of these factors indicate that dogs are good sentinels for assessing the risk of human infection with JEV [57,58]. Therefore, we developed a convenient, rapid, sensitive, and specific ICS kit for JEV seroconversion surveillance in dogs.

The ICS is based on the JEV recombinant EDⅢ protein, which conjugates with colloidal gold to produce an immunogold complex, and it is capable of a more rapid and sensitive diagnosis and monitoring JEV antibodies in dog sera. JEV envelope protein domain 3 (EDⅢ) harbors the antigenic determinant that is responsible for eliciting neutralizing antibodies [59]. In this study, the recombinant EDⅢ protein conjugated with colloidal gold could bind to JEV antibodies in dog sera, and the binding antibodies were captured by immobilized SPA to form a red band, indicating the presence of JEV antibodies in the samples.

Previous studies have reported that various serological methods, such as VN, ELISA, and HI, can be used for JEV antibody surveillance in dogs [17,29,60]. However, these methods are expensive; time-consuming; and require skilled persons, special equipment, well-developed labs, and a live virus. Our developed ICS kit is easy to perform, and the results can be obtained within 15 min (Figure 3). Furthermore, we tested 586 dog sera samples collected under field conditions and showed that the developed ICS kit has a high specificity and sensitivity. These results are comparable with those of previously developed ICS kits used for JEV detection in pigs or for the detection of other canine virus antibodies [36,61,62].

The dog is one of the most important companion animals for human beings. With the development of urbanization, people often keep dogs as companions, and contact between dogs and humans is increasing and becoming worthwhile, as it brings joy. A total of 586 dog sera samples were collected from numerous pet immunization centers, hospitals, farms, and abandoned dog shelters of different districts of Shanghai and tested with the ICS method, and 179 samples were positive for JEV antibodies. A 29.98% JEV-antibody-positive rate was found, which indicates a high JEV infection rate in Shanghai. A study conducted in Japan showed that 25% of dogs have high JE virus-neutralizing antibodies, with relatively high seropositivity detected in the Shikoku (61%) and Kyushu (47%) districts of western Japan [19]. In another study conducted in a Cambodian village, a high JEV seroprevalence of 35% was detected in dogs [15]. Humans and pet dogs live in the same area and do not receive JEV vaccines; therefore, the JEV-antibody-positive sera from dogs suggest that mosquitoes carry JEV in human environments, indicating that further virome surveillance of local mosquitoes is required [63].

Shanghai is located near the sea and has a subtropical climate, higher temperatures (20~31 °C), and high rainfall and humidity, which is suitable for mosquito breeding [46,47]. The presence of higher mosquito vectors shows that JEV can easily spread during the mosquito breeding season (summer—June to September) [47]. In the present investigation, we also found an association between the JE epidemic season (August to October) and the positive rate of JEV antibodies in dogs. After the epidemic season, the positive rate of JEV antibodies significantly increased from summer (21.5%) to autumn (46.3%) as compared to the previous epidemic season (the average positive rate was 16.4% during March and April). This proves that JEV infection in dogs is seasonal in China.

In conclusion, our data indicate that the prevalence of JEV is relatively high in Shanghai, China. The ICS was developed for the detection of JEV antibodies in dogs, and the assay was applied in a serological survey on JEV infection to assess the risk of JEV infection to humans.

## Figures and Tables

**Table 1:** Comparison of the results between ICS and virus neutralization among dogs in Shanghai, China.

ICS	NT		Total
	Positive	Negative	
Positive	172	7	179
Negative	13	394	407
Total	185	401	586

The coincidence ratio of ICS to NT is 96.6%.

**Table 2:** Seroprevalence of JEV determined using ICS in dogs in Shanghai, China.

Variable		Category	No. Examined	No. Positive	Positive Rate
Season	Spring	March	32	8 (25.0%)	16.4% ^a^
		April	41	6 (14.6%)	
		May	52	5 (9.6%)	
	Summer	June	56	10 (17.9%)	21.5% ^a^
		July	63	15 (23.8%)	
		August	57	13 (22.8%)	
	Autumn	September	53	22 (41.5%)	46.3% ^b^
		October	71	36 (50.7%)	
		November	60	28 (46.7%)	
	Winter	December	30	12 (40.0%)	35.7% ^b^
		January	33	10 (30.3%)	
		February	38	14 (36.8%)	
LivingEnvironment		Domestic dogs	355	73	20.6% ^a^
		Breeding dogs	134	58	43.3% ^b^
		Stray dogs	97	48	49.5% ^b^
Total			586	179	29.98%

Values with the same superscript (a) letters showed no statically significant difference (*p* > 0.05). However, those with different superscript (b) letters showed a statically significant difference to others (*p* < 0.05).

## Data Availability

No new data were created or analyzed in this study. Data sharing is not applicable to this article.
